# Food-Derived Antihypertensive Peptides: Mechanisms, Multi-Methodological Approaches, Bioavailability, and Functional Food Applications

**DOI:** 10.3390/molecules31101648

**Published:** 2026-05-13

**Authors:** Lucía Castillejos Ordóñez, Nathaly Marcela Guzmán Pineda, Beatriz Isabella Encalada Lizcano, Astrid Carolina Lugo Díaz, Luis Jorge Corzo Ríos, Cristian Jimenez Martínez, Jorge Carlos Ruiz Ruiz

**Affiliations:** 1 Escuela de Biotecnología, Universidad Anáhuac Mayab, Carr. Mérida Progreso Km. 15.5 AP. 96 Cordemex, Mérida C.P. 97308, Yucatán, Mexico; lucia.castillejos@anahuac.mx (L.C.O.); nathaly.guzman@anahuac.mx (N.M.G.P.); 00453573@anahuacmayab.edu.mx (B.I.E.L.); 2 Escuela de Nutrición, Universidad Anáhuac Mayab, Carr. Mérida Progreso Km. 15.5 AP. 96 Cordemex, Mérida C.P. 97308, Yucatán, Mexico; astrid.lugo@anahuac.mx; 3 Unidad Profesional Interdisciplinaria de Biotecnología, Instituto Politécnico Nacional, Av. Acueducto S/N, Col. Barrio La Laguna Ticomán, Ciudad de México C.P. 07340, Mexico; lcorzo@ipn.mx; 4 Escuela Nacional de Ciencias Biológicas, Instituto Politécnico Nacional (IPN), Av. Wilfrido Massieu S/N, Unidad Profesional Adolfo López Mateos, Zacatenco, Alc. Gustavo A. Madero, Ciudad de México C.P. 07738, Mexico; cjimenezh@ipn.mx; 5 Tecnológico Nacional de México, Campus Progreso, Boulevard Tecnológico de Progreso S/N x 62, Progreso C.P. 97320, Yucatán, Mexico

**Keywords:** antihypertensive peptides, ACE inhibition, bioavailability, molecular stability, functional foods, food-derived bioactives

## Abstract

This systematic review was conducted and reported according to the PRISMA 2020 statement to synthesize evidence published between January 2020 and January 2025 on food-derived antihypertensive peptides, with emphasis on mechanisms of action, molecular stability, bioavailability, and functional food applications. PubMed, Scopus, and Web of Science were searched using combined terms related to bioactive or ACE-inhibitory peptides, stability or bioavailability, and alternative protein sources. Original peer-reviewed studies in English evaluating antihypertensive or ACE-inhibitory peptides from plant, marine, insect, fungal, dairy, or terrestrial animal matrices were considered eligible when they reported experimental evidence on activity, stability, transport, or in vivo efficacy. Three reviewers independently screened records and extracted data. A total of 177 studies were included. Plant and marine matrices accounted for approximately 72% of the evidence base, with a strong focus on low-molecular-weight peptides (<3 kDa) and multistage validation pipelines integrating in silico screening, in vitro enzymatic assays, Caco-2 transport models, ex vivo assays, and spontaneously hypertensive rat studies. Overall, the evidence supports the antihypertensive potential of selected food-derived peptides, particularly through ACE inhibition and related vascular mechanisms. Encapsulation and advanced delivery approaches improved peptide stability and bioavailability in several studies. Food-derived antihypertensive peptides represent promising candidates for functional foods and nutraceuticals; however, greater methodological standardization, formal risk-of-bias assessment in primary studies, and well-designed human trials remain necessary to strengthen translation into practice.

## 1. Introduction

Hypertension (HTN) is one of the most widespread chronic diseases worldwide and remains a leading modifiable risk factor for cardiovascular morbidity and mortality. According to the World Health Organization, more than one billion people are currently affected, with prevalence exceeding 20% in adults and disproportionately impacting low- and middle-income countries [1,2]. Despite major advances in pharmacotherapy, global awareness, diagnosis, and long-term adherence to antihypertensive treatment remain insufficient. This is particularly concerning given the strong association between HTN and arteriosclerosis, stroke, heart failure, and renal disease [1]. Blood pressure regulation is governed by several physiological pathways, among which the renin–angiotensin–aldosterone system (RAAS) plays a central role. The angiotensin-converting enzyme (ACE) is a key mediator of RAAS activation and has become a primary therapeutic target for HTN management [3]. Synthetic ACE inhibitors—such as captopril, enalapril, and lisinopril—are widely prescribed; however, their use is frequently associated with adverse effects including dry cough, dizziness, headache, fatigue, and hyperkalemia, which reduce treatment compliance [3,4]. These limitations have stimulated growing interest in naturally occurring ACE-inhibitory peptides derived from food proteins as complementary or alternative antihypertensive agents. Over the past decade, research on food-derived bioactive peptides has expanded substantially, reflecting advances in extraction technologies, peptidomics, and in silico prediction tools. Marine organisms, plant by-products, dairy proteins, and edible insects have emerged as promising sources of low-molecular-weight peptides with demonstrated bioactivities, including antioxidant, immunomodulatory, antihyperglycemic, antimicrobial, and antihypertensive effects [5,6,7]. However, despite substantial evidence supporting ACE inhibition in vitro, there remains debate regarding the extent to which these peptides remain stable during gastrointestinal digestion, reach systemic circulation, and exert physiological effects in vivo. Some studies indicate limited bioavailability of certain peptides due to rapid enzymatic degradation [8], whereas others report measurable antihypertensive responses in animal models and human trials [9], highlighting ongoing scientific debate. Recent efforts increasingly integrate multistage methodological approaches—combining in silico screening, enzymatic assays, Caco-2 transport models, and spontaneously hypertensive rat (SHR) studies—to improve prediction of peptide efficacy and stability. Encapsulation technologies have also gained attention as a strategy to enhance bioavailability and functional incorporation into foods [10]. Nevertheless, critical gaps remain in standardization, clinical validation, and translation to real-world food systems. The present review synthesizes scientific evidence published between 2020 and 2025 on food-derived antihypertensive peptides, examining their mechanisms of action, stability, bioavailability, and potential application in functional foods and nutraceuticals. We highlight emerging trends, knowledge gaps, and areas of scientific divergence, and we conclude by proposing future research directions needed to advance these peptides toward regulatory approval and consumer-ready formulations.

## 2. Results

This review synthesizes the most recent evidence (2020–2025) on food-derived anti-hypertensive peptides and demonstrates that this area of research is both dynamic and rapidly expanding. The findings consistently confirm the capacity of these peptides to modulate blood pressure—primarily through ACE inhibition—while also underscoring the growing interest in novel and underutilized protein sources. The valorization of food by-products and processing waste has emerged as a particularly promising strategy for generating bioactive peptides suitable for nutraceutical development. Moreover, the increasing focus on molecular stability, gastrointestinal resistance, and bioavailability reflects a maturation of the field toward translational applications. Collectively, the diversity of mechanisms of action, the strengthening evidence for their functional performance, and their potential incorporation into functional foods highlight the relevance of these peptides as complementary tools for the prevention and management of hypertension.

### 2.1. Food Sources of Antihypertensive Peptides

**Figure 1:**
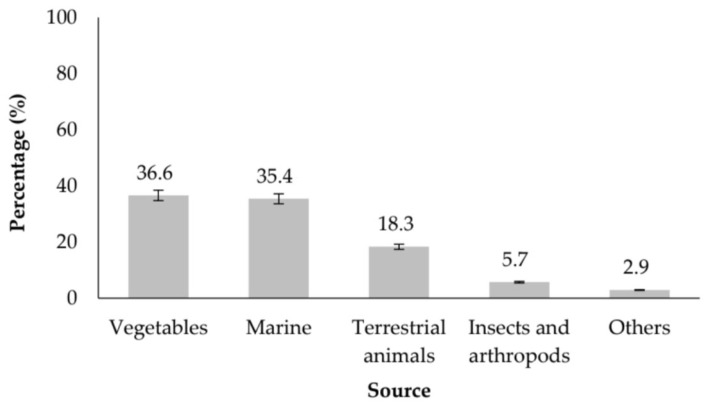
Proportion of antihypertensive peptide studies by source category (2020–2025).

The study-selection process is summarized in the PRISMA 2020 flow diagram (Figure 1). After database searching, deduplication, title/abstract screening, and full-text eligibility assessment, 177 studies were included in the qualitative synthesis. Analysis of these 177 articles reveals a broad and evolving landscape of food sources investigated for antihypertensive peptide production. The distribution of sources demonstrates a strong emphasis on alternative and innovative protein matrices, reflecting global efforts to diversify nutraceutical ingredients and valorize underutilized resources.

Vegetable-derived peptides constitute the largest category (36.6%, n = 64), supported by extensive research on legumes, cereals, and oilseed by-products. Marine organisms represent the second most studied group (35.4%, n = 62), with work frequently focusing on fish by-products, algae, and seaweed—matrices known for their high protein quality and sustainability potential. Together, plant and marine sources account for more than two-thirds (72%) of all included studies, underscoring their central role in the development of ACE-inhibitory peptides.

Terrestrial animal sources comprise 18.3% (n = 32) of publications, largely driven by studies on milk, eggs, and meat-derived proteins. Although these are well-established substrates for bioactive peptide generation, their relative proportion in recent literature suggests a shift in interest toward more sustainable alternatives.

Emerging protein categories—including insects and arthropods (5.7%, n = 10) and fungi (1.1%, n = 2)—show increasing scientific attention despite their limited representation. These matrices offer unique amino acid profiles and hold considerable promise for future peptide-based functional ingredients, particularly within circular bioeconomy frameworks. The full distribution of sources is presented in Figure 1, highlighting the diversity and growing innovation in antihypertensive peptide research.

#### 2.1.1. Vegetable Sources

**Figure 2:**
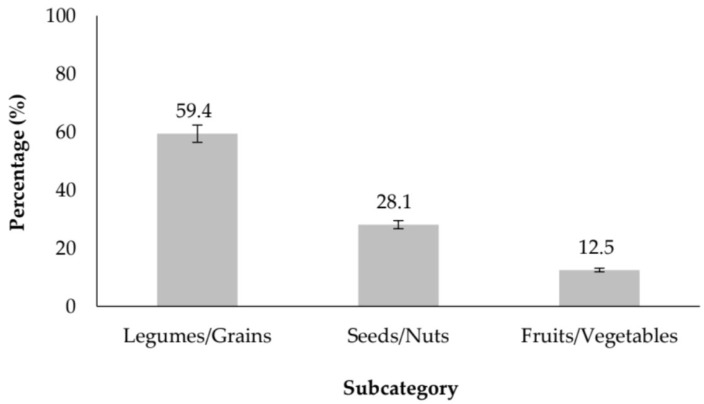
Distribution of Peptide Sources Among Vegetable Subcategories (2020–2025).

Vegetable matrices represent the largest category identified in this review, accounting for 64 of the included studies (Figure 2). Within this group, research is strongly concentrated on legumes and grains, which comprise 59.4% of all vegetable-derived peptide studies. This emphasis reflects their high protein content, balanced amino acid composition, and demonstrated efficiency in releasing bioactive peptides through enzymatic hydrolysis or fermentation processes [4,5,6]. Notably, studies on fermented soybean products [5,7] and rice-derived peptides [7,8] consistently report enhanced ACE-inhibitory activity following microbial or enzymatic modification, supporting the technological relevance of these substrates. Seeds and nuts constitute the second most represented subcategory (28.1%), driven by increasing interest in oilseed by-products and their valorization within circular bioeconomy frameworks. Representative examples include hemp seed proteins [8], pumpkin seed concentrates [9], and other oilseed meals such as sunflower or rapeseed [10], all of which have yielded peptides with measurable antihypertensive potential. In contrast, fruits and vegetable-derived proteins remain underexplored, representing only 12.5% of studies. Despite their lower frequency, recent investigations—including peptides isolated from tea residues [11] and *Ziziphus jujuba* fruit [12]—suggest growing diversification toward plant sources traditionally overlooked in protein research. These emerging matrices may provide unique peptide profiles and warrant further investigation.

#### 2.1.2. Marine Sources

**Figure 3:**
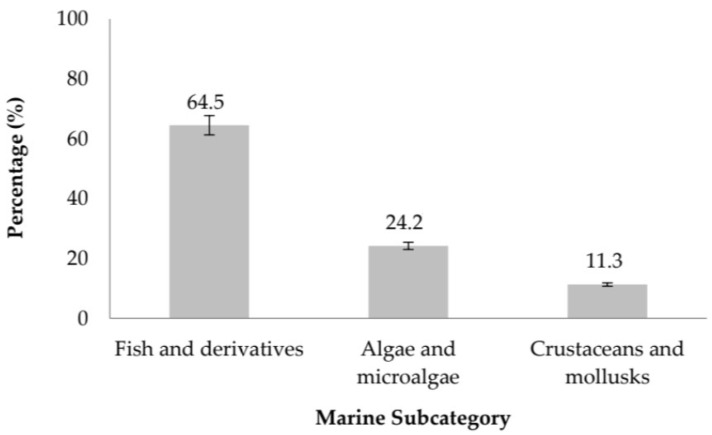
Distribution of Peptide Sources Among Marine Subcategories.

Marine matrices constitute the second most prolific category in this review, contributing studies to the dataset (Figure 3). Within this group, fish and fish-derived materials overwhelmingly dominate, representing 64.5% of all marine-source investigations. This strong emphasis reflects the continued interest in valorizing byproducts from the fishing and aquaculture industries, including fish skin gelatin [13] and diverse fish protein hydrolysates generated from processing residues [14,15]. The high protein content and favorable amino acid composition of these substrates make them particularly suitable for the production of ACE-inhibitory peptides. A notable emerging trend is the increasing exploration of algae and microalgae, which account for 24.2% of marine-source studies. These organisms are gaining global recognition as sustainable, renewable, and high-yield biomass sources, contributing both nutritional and environmental value. Research in this category includes work on *Chlorella pyrenoidosa* [16,17] as well as a wide range of bioactive peptides derived from edible seaweeds [18]. Their growing prominence aligns with broader efforts to develop climate-resilient protein alternatives. In contrast, crustaceans and mollusks represent a smaller proportion of the literature (11.3%). Although less frequently studied, notable examples include peptides obtained from sea cucumber (*Stichopus japonicus*) [1] and blue mussel [19], both of which have shown promising antihypertensive potential. Despite their lower representation, these sources offer unique peptide profiles and may warrant further exploration in future research.

#### 2.1.3. Protein Composition Relevant to the Generation of Bioactive Peptides

(a) The antihypertensive potential of food-derived peptides is fundamentally determined by the biochemical characteristics of their precursor proteins, the specific peptide sequences released, and the efficiency of delivery systems. The predominant mechanism of action across studies is ACE inhibition, which is strongly influenced by the amino acid residues occupying the C-terminal P1, P1′, and P2′ positions of the peptide chain [20,21].
(b) Hydrophobic Residues. A high abundance of hydrophobic amino acids—such as Pro, Phe, Tyr, Trp, Leu, Ile, and Val—in the parent protein is a key determinant of bioactivity. These residues are frequently located at the C-terminus of the most potent ACE-inhibitory peptides and enhance binding affinity to the ACE active site. This biochemical pattern explains the extensive research interest in legumes and grains, whose storage proteins (e.g., globulins and albumins) naturally contain elevated levels of these hydrophobic residues [22,23].
(c) Proline Content. Peptides enriched in proline exhibit remarkable resistance to gastrointestinal proteolysis, which increases their stability during digestion and consequently improves their physiological bioavailability [24]. This feature is especially prominent in peptides derived from milk proteins (e.g., caseins) within terrestrial animal sources, as well as in marine collagens from fish and related derivatives [25].
(d) Molecular Weight. Across all food matrices, antihypertensive peptides typically fall within a low molecular weight range (1 kDa to 3 kDa). Such small peptides, documented widely in marine [21] and vegetable sources [8], are more readily absorbed in the gastrointestinal tract and demonstrate enhanced ACE-inhibitory efficiency due to superior diffusion and interaction with ACE catalytic sites.

Collectively, these biochemical patterns indicate that source selection is not merely dictated by availability or food system relevance. Rather, it reflects a targeted, rational approach to choosing proteins with favorable amino acid profiles that, upon enzymatic or microbial processing, can yield stable, low-molecular-weight, hydrophobic-rich peptides with optimal antihypertensive activity.

### 2.2. In Silico, In Vitro, Ex Vivo, and In Vivo Assays for Antihypertensive Peptides

The validation of antihypertensive peptides derived from novel food sources increasingly relies on multi-stage analytical and biological approaches. Recent studies employ a combination of in silico, in vitro, ex vivo, and in vivo assays to systematically identify, characterize, and evaluate peptide bioactivity, stability, and safety. Advanced analytical techniques, such as Liquid Chromatography–Mass Spectrometry (LC-MS/MS) [9,26,27,28] and SDS-PAGE profiling [2,29,30,31,32,33], are commonly used to determine peptide composition, molecular weight distribution, and purity prior to bioactivity assessment. These tools provide essential information for elucidating peptide structure–function relationships and guiding the selection of candidates for further biological testing.

By integrating computational prediction, biochemical assays, cell-based evaluations, and animal models, these studies create a comprehensive validation pipeline that strengthens the evidence supporting the antihypertensive potential of each peptide. This methodological progression—from molecular identification to functional biological assessment—helps establish a peptide’s therapeutic promise, possible mechanisms of action, and suitability as a functional food ingredient, nutraceutical component, or pharmaceutical lead. The specific combination of assays employed varies according to the aims of each study, but the multi-tiered structure remains a unifying feature across recent literature. Accordingly, the methodological value of multistage pipelines lies not in treating all assays as equivalent, but in integrating complementary levels of evidence, from computational prioritization to biochemical testing and physiological validation.

#### 2.2.1. In Silico Assays for Antihypertensive Peptides

In silico methodologies serve as predictive and screening tools for the evaluation of these molecules. These approaches can prioritize candidate peptides based on binding affinity, simulated gastrointestinal stability, and predicted safety-related properties, but they do not constitute biological validation on their own. In silico methodologies serve as predictive and screening tools for the evaluation of these molecules. They can prioritize candidate peptides on the basis of binding affinity, simulated gastrointestinal stability, and predicted safety-related properties, but they do not constitute biological validation on their own. These approaches include molecular docking and virtual screening to assess peptide binding affinity to ACE, simulating gastrointestinal digestion to predict the peptide stability when is consumed orally and the properties of the released fragments, and bioinformatic evaluation of activity prediction, toxicity, allergenicity, and drug-likeness evaluation with tools such as PeptideRanker, BIOPEP-UWM, and SwissADME, which offer a cost-effective and time-efficient alternative to experimental trials (Table 1).

In silico methodologies have become essential tools for the preliminary evaluation of antihypertensive peptides. These computational approaches provide a rapid, cost-effective means to predict peptide bioactivity, stability, and safety before undertaking more resource-intensive laboratory experiments.

Several key strategies are employed:

(a) Molecular Docking and Virtual Screening: These methods assess the binding affinity of peptides to the ACE active site, enabling the identification of promising candidate sequences with high inhibitory potential.
(b) Simulated Gastrointestinal Digestion: In silico digestion models predict peptide stability during oral consumption, as well as the nature and bioactivity of digestion-derived fragments. This step helps anticipate whether peptides can withstand enzymatic degradation and remain functionally intact in vivo.
(c) Bioinformatic Activity Prediction: Tools such as PeptideRanker, BIOPEP-UWM, and SwissADME evaluate various parameters including bioactivity probability, toxicity, allergenicity, physicochemical properties, and drug-likeness. These analyses help filter out peptides with undesirable characteristics early in the research pipeline.

To investigate peptides derived from *Protaetia brevitarsis*, several studies employed molecular docking analyses, which revealed strong hydrogen-bonding interactions between these peptides and key residues within the ACE active sites [22,30,31,32,33]. Other works similarly confirmed competitive inhibition mechanisms, supporting the potential of insect-derived peptides as ACE inhibitors [2,31,34]. In parallel, studies on Sophia and Camelina protein hydrolysates combined PeptideRanker, BIOPEP, and in silico simulated digestion to predict bioactivity while simultaneously assessing toxicity and allergenicity—an essential step for ensuring peptide safety in nutraceutical applications [30,32,35]. Additional research used docking and virtual screening to identify structurally potent ACE-inhibitory candidates from quinoa and fish protein hydrolysates [28,30,32,33]. In several of these works, peptide stability, digestibility, and multifunctionality were further validated by integrating in silico gastrointestinal digestion models with physicochemical property predictions, strengthening the evidence for their antihypertensive potential [28,30,32,33].

Together, these computational tools offer a robust, high-throughput screening platform that complements experimental work and significantly accelerates the discovery process.

#### 2.2.2. In Vitro Assays for Antihypertensive Peptides

In vitro assays serve as an essential first step for validating the biological activity of antihypertensive peptides under controlled laboratory conditions before progressing to ex vivo or in vivo models. These methods primarily evaluate ACE inhibitory activity, antioxidant potential, cytotoxicity, and bioavailability, offering a detailed functional profile of peptide candidates (Table 2). The ACE inhibitory assay remains the most widely used technique, quantifying a peptide’s capacity to block angiotensin-converting enzyme activity. To complement ACE inhibition data, antioxidant assays—including DPPH, ABTS, FRAP, and ORAC—are frequently employed to assess the ability of peptides to neutralize free radicals and mitigate oxidative stress, which is closely linked to hypertension pathology.

Safety and cytocompatibility are typically evaluated using MTT assays in endothelial or intestinal cell lines (e.g., HUVECs, Caco-2). Additional functional insights are gained through NO production assays, which reveal vasodilatory potential, and Western blotting, which is used to analyze key signaling pathways such as Akt/eNOS involved in vascular homeostasis. Several studies exemplify the combined application of these methods. Surimi-derived peptides were evaluated for ACE inhibition and NO production in HUVECs [28,33,36]. *Lupinus mutabilis* hydrolysates were assessed through ACE inhibition and DPPH radical-scavenging assays [37,38]. Other investigations examined the effects of physical processing on ACE inhibitory and antioxidant properties [27,37]. Similarly, peptides from alternative protein sources were tested for ACE and DPP-IV inhibition along with antioxidant activity using DPPH, TEAC, and FRAP assays [26,27]. To predict intestinal absorption, multiple studies incorporated Caco-2 monolayer transport models, providing insight into peptide permeability and potential oral bioavailability [26,27,39,40]. Collectively, these in vitro strategies offer essential information on peptide functionality, stability, and safety, forming a robust foundation for subsequent ex vivo and in vivo validation.

**Table 2:** Summary of In Vitro Methodologies Used for the Evaluation of Antihypertensive Peptides.

In Vitro Assay	Purpose	Articles
ACE Inhibitory Assay	Measures ability of peptides to inhibit angiotensin-converting enzyme (ACE), key for antihypertensive effect	[17,30,33,34,35]
DPPH Assay Radical Scavenging	Evaluates antioxidant potential by measuring free radical scavenging	[17,33,35,36]
ABTS/ORAC Assays	Quantifies antioxidant capacity against ABTS•+ and peroxyl radicals	[35,41]
Cell Viability/MTT Assay	Assesses cytotoxicity and safety of peptides in cell cultures	[1,7]
Caco-2 Cell Monolayers	Simulates intestinal absorption and bioavailability	[7,17]
Western Blot (Akt/eNOS Pathway)	Evaluates vasodilatory mechanisms via phosphorylation signaling	[32]
NO Production Assay	Measures nitric oxide release in endothelial cells for vascular health	[1,32]

#### 2.2.3. Ex Vivo Assays for Antihypertensive Peptides

Ex vivo assays assess the physiological effects of bioactive peptides on isolated tissues and vascular systems, serving as a crucial intermediary between in vitro biochemical tests and in vivo whole-organism studies. These methodologies enable researchers to evaluate how peptides influence vascular function—particularly vasodilation and endothelial responsiveness—in biological tissues that retain their native structural and functional characteristics. In these studies, aortic rings or primary vascular endothelial cells are commonly isolated and exposed to peptides to measure functional outcomes such as nitric oxide (NO) production, vascular relaxation, and modulation of endothelial signaling pathways (Table 3).

**Table 3:** Summary of Ex Vivo Methodologies Used to Evaluate Antihypertensive Peptides.

Ex Vivo Assay	Purpose	Articles
Vasodilation Effect (Aortic rings)	Measures response vessels relaxation of isolated	[31]
NO Production in Vascular Tissue	Evaluates endothelial function and vasodilatory potential	[1,32]
ACE Activity in Tissue Extracts	Tests antihypertensive effect beyond cell culture	[31]

For example, activation of the Akt/eNOS pathway in endothelial cells has been used to elucidate mechanisms underlying peptide-induced vasodilation [40,42]. Similarly, other studies have reported enhanced NO release and improved vascular relaxation responses following treatment with antihypertensive peptides [2,34]. By providing functional readouts under near-physiological conditions, ex vivo assays offer critical insights into the bioactive potential of peptides and help validate mechanistic hypotheses prior to advancing to in vivo animal models. These approaches thus represent an essential component of the stepwise evaluation framework for antihypertensive peptide research.

#### 2.2.4. In Vivo Assays for Antihypertensive Peptides

In vivo models provide the most physiologically relevant evidence for the antihypertensive potential of food-derived peptides (Table 4). Among these, spontaneously hypertensive rats (SHRs) are the gold standard for evaluating peptide efficacy due to their similarity to human essential hypertension. These assays enable researchers to monitor direct cardiovascular outcomes, systemic effects, and safety profiles following peptide administration. In several studies, systolic blood pressure (SBP), diastolic blood pressure (DBP), and mean arterial pressure (MAP) were measured over time to determine treatment efficacy [43,44,45,46]. Dose-dependent reductions in blood pressure have been consistently reported, with high-dose peptides producing reductions of up to 42–47 mmHg in SBP [43]. In dexamethasone-induced hypertensive rats, peptide administration also resulted in significant SBP decreases, demonstrating their effectiveness across different hypertension models [46]. Beyond blood pressure metrics, in vivo assays commonly include evaluations of body weight, organ coefficients, and overall health status to assess systemic toxicity. Multiple studies reported no adverse effects on growth or organ size, supporting the safety of peptide interventions [43,47]. Histological analyses provide additional insights into physiological restoration. Peptide-treated animals exhibited improvements such as normalized cardiac morphology, reduced renal edema, and attenuation of tissue damage associated with hypertension [43,46]. Mechanistic investigations often focus on Renin–Angiotensin System (RAS) modulation, a key regulatory pathway in blood pressure control. Studies demonstrated that anti-hypertensive peptides can rebalance the RAS by upregulating the protective ACE2–Ang (1–7)–Mas axis while downregulating the vasoconstrictive ACE–AngII–AT1 axis [43,44,45]. This dual regulatory effect aligns with the mechanisms of clinically used ACE inhibitors and supports therapeutic relevance. Oxidative stress biomarkers further clarify peptide activity. Measurements of lipid peroxidation (LPO), superoxide dismutase (SOD), and glutathione (GSH) indicate that peptide treatment reduces oxidative stress and enhances endogenous antioxidant defenses. For instance, peptide-treated groups showed lower LPO levels and increased SOD and GSH activity, reflecting improved vascular health and reduced hypertensive damage [46]. Taken together, in vivo studies provide strong evidence for the efficacy, safety, and multifaceted mechanisms of antihypertensive peptides, validating their potential for functional food and nutraceutical applications.

### 2.3. Molecular Stability and Bioavailability of Bioactive Peptides

The successful translation of in vitro bioactivity into in vivo therapeutic efficacy for food-derived peptides depends largely on their molecular stability and bioavailability. Peptides must withstand multiple harsh conditions—from gastrointestinal (GI) digestion to systemic transport—while maintaining their structural integrity and functional activity. Major challenges include enzymatic degradation, instability during food processing, chemical modification, and limited absorption across the intestinal epithelium [39,40]. This section summarizes current experimental evidence from alternative protein sources that address these critical factors.

#### Gastrointestinal Stability and Resistance to Enzymatic Degradation

The gastrointestinal tract represents one of the most significant barriers to the efficacy of orally consumed peptides. Exposure to proteolytic enzymes such as pepsin, trypsin, and chymotrypsin can rapidly degrade peptides before absorption occurs. To assess this, simulated gastrointestinal digestion (SGD) models are widely employed as an initial screening tool (Figure 1). Outcomes vary substantially: while some peptides are completely hydrolyzed, others demonstrate strong resistance and may even exhibit enhanced bioactivity after digestion.

Resistance to gastrointestinal proteolysis depends not only on peptide amino acid composition, but also on the structural context of the peptide within the parent protein, including conformational accessibility, local flexibility, and the extent to which cleavage sites are exposed or sterically protected from enzymatic attack (Table 4). However, resistance to gastrointestinal proteolysis depends not only on peptide amino acid composition, but also on the structural context of the peptide within the parent protein, including conformational accessibility, local flexibility, and the extent to which cleavage sites are exposed or sterically protected from enzymatic attack. Resistance to gastrointestinal proteolysis is influenced by both peptide sequence and the structural context of the parent protein. Although Pro-rich motifs are often associated with reduced susceptibility to enzymatic cleavage, peptide persistence during digestion also depends on factors such as protein conformation, regional mobility, and steric accessibility of protease-sensitive sites. Therefore, sequence-based predictions should be interpreted together with experimental digestion data [49]. In another study, the peptide PMYGGGMV preserved both ACE-inhibitory and antioxidant activity after digestion, indicating a robust structural profile compatible with oral delivery [50]. However, peptide stability varies widely across sources (Table 4). In black cricket (*Gryllus assimilis*) protein hydrolysates, ACE-inhibitory activity was completely lost after SGD, although antioxidant activity remained stable or improved [49]. This highlights the heterogeneous stability of peptides within a single hydrolysate and underscores the importance of performing post-digestion functional assays. Conversely, protein hydrolysates derived from spent hen retained strong ACE-inhibitory activity following both pepsin and pancreatin digestion [51], demonstrating that outcomes can differ markedly across substrates. To exert systemic antihypertensive effects, peptides must cross the intestinal barrier and enter the bloodstream. Caco-2 cell monolayers remain the gold standard in vitro model for predicting intestinal permeability and peptide transport mechanisms. One of the most relevant transport systems is PepT1, a proton-dependent transporter that preferentially absorbs dipeptides and tripeptides [52].

Studies using Caco-2 models have shown that peptides from spent hen hydrolysates exhibit favorable permeability profiles, consistent with their reported antihypertensive activity in spontaneously hypertensive rats (SHRs) [51]. These findings suggest that GI stability combined with transepithelial transport efficiency may be a stronger predictor of in vivo antihypertensive outcomes than in vitro ACE inhibition alone.

Once absorbed, peptides must resist degradation within the bloodstream to exert physiological effects. For instance, peptides from duck blood protein remained intact under simulated blood plasma protease conditions, indicating a high potential for systemic stability [53]. Complementary ex vivo data from fermented milk peptides revealed a bioavailability of 7.9%, demonstrating that a measurable proportion of active peptides successfully reach circulation [54].

In addition to cardiovascular outcomes, peptide stability influences nutrient transport across the intestinal barrier. The PMYGGGMV–ferrous chelate, for example, showed enhanced iron transport across Caco-2 monolayers due to its strong metal-chelation capacity and resistance to GI degradation [50], illustrating how peptide stability can extend beyond antihypertensive functions to broader nutritional applications.

### 2.4. Functional Food Applications and Industrial Potential

The incorporation of antihypertensive peptides into functional foods represents one of the most promising strategies for translating laboratory findings into accessible public health solutions. This application route leverages everyday dietary habits, enabling consumers to obtain cardiovascular benefits through foods they already consume. However, successful implementation requires careful consideration of technological feasibility, sensory quality, safety, and industrial scalability.

#### 2.4.1. Technological and Sensory Considerations in Functional Foods

Two major strategies have been identified for incorporating bioactive peptides into food systems:

(a) Direct incorporation of peptide hydrolysates. Hydrolysates from sources such as *Lupinus mutabilis* [55,56], corn and soy proteins [49,50], and fish by-products [51] have been evaluated as functional ingredients. Despite promising bioactivity, the sensory impact—particularly bitterness associated with hydrophobic amino acids—remains a major barrier to consumer acceptance. As a result, many studies emphasize the optimization of hydrolysis conditions and enzyme selection to minimize bitterness while preserving ACE-inhibitory capacity. Achieving this balance is essential for the wide adoption of peptide-enriched foods.
(b) In situ peptide generation during food processing. Fermentation and enzymatic processing offer a second, highly effective strategy for releasing antihypertensive peptides directly within the food matrix. Examples include:
  - Fermentation of soy with Enterococcus faecium, significantly enhancing ACE-inhibitory activity [5];
  - Fermentation of rice with targeted bacterial strains [51,52,53];
  - Cheese ripening with Lactobacillus helveticus, known to naturally produce ACE-inhibitory peptides during maturation [24].

This approach seamlessly integrates bioactive peptide formation into traditional manufacturing practices, making it especially promising for dairy and fermented plant-based products.

#### 2.4.2. Safety and Regulatory Requirements

Safety assessment is central to the commercialization of peptide-containing functional foods. While most studies employ food-grade enzymes and GRAS microorganisms [5,24], ensuring consumer safety demands further evaluation of:

- Allergenicity, particularly for peptides derived from soy, dairy, nuts, or crustaceans;
- Toxicological profiles, including cytotoxicity and gastrointestinal compatibility;
- Stability during storage, which affects both safety and efficacy.

Hydrolysis may reduce allergenicity in some cases, but this effect must be validated experimentally rather than assumed.

Regulatory pathways, such as FDA GRAS approval or EU Novel Food authorization, require extensive scientific documentation on safety, stability, efficacy, and manufacturing reproducibility. These steps are seldom addressed in research publications, yet they form a pivotal bottleneck between laboratory success and industrial adoption.

#### 2.4.3. Commercial and Technological Potential

The reviewed literature reveals a strong and growing interest in the industrial feasibility and economic potential of food-derived antihypertensive peptides. A central trend is the valorization of food industry by-products, such as fish skins and bones [54,57] and plant processing residues [11]. These substrates provide low-cost, sustainable raw materials, aligning with global priorities for circular economies and waste minimization. Several studies discuss pilot-scale or industrial-scale production, suggesting that peptide-based ingredients are beginning to move beyond the research phase into commercial development [54,57]. Their advancement is supported by innovative technologies, including:

- High hydrostatic pressure (HHP) to improve enzymatic hydrolysis efficiency and peptide yield [47];
- Advanced fermentation strategies to enhance peptide release and reduce bitterness [58];
- Nanoencapsulation systems to stabilize peptides and improve their delivery within food matrices.

Although patents were not explicitly reported, the novelty of peptide sequences and the growing sophistication of extraction, hydrolysis, and formulation methods indicate substantial intellectual property opportunities.

#### 2.4.4. Integrated Outlook

Together, technological innovation, sensory optimization, regulatory compliance, and industrial scalability define the multidimensional pathway required to bring antihypertensive peptides from bench to market. Functional foods enriched with bioactive peptides hold strong potential to reshape preventive nutrition and cardiovascular health, provided that future research continues to bridge the critical gaps between bioactivity, stability, consumer acceptance, and commercial feasibility. This systematic review (2020–2025) demonstrates that food-derived antihypertensive peptides constitute a robust and promising alternative for the prevention and management of hypertension, offering a superior safety profile compared with classical synthetic ACE inhibitors. The evidence highlights a clear shift in the field toward sustainable resource utilization and coproduct revalorization, reflected in the predominance of plant- and marine-derived peptides, which together account for 72% of the studies included. These sources provide proteins with favorable characteristics—high hydrophobicity, proline-rich motifs, and low molecular weight—that contribute to enhanced ACE inhibition and improved physiological stability.

### 2.5. Emerging Trends

Recent research trajectories highlight a marked shift toward marine resources and plant-based by-products, driven by the search for sustainable, cost-effective alternatives to conventional protein inputs. This shift aligns with global priorities for environmental stewardship, circular bioeconomy strategies, and reduced reliance on resource-intensive animal-derived proteins.

One of the most significant developments is the revalorization of food waste as a raw material for peptide production. Industrial by-products such as fish skin, fish viscera, and processing trimmings have been successfully transformed into peptide-rich hydrolysates with demonstrable ACE-inhibitory activity. Similarly, agricultural residues such as defatted lemon basil seed meal illustrate the growing interest in repurposing materials previously considered low-value or waste.

This emerging paradigm reflects a systems-level approach: peptide research is increasingly situated within the broader context of human health, environmental sustainability, economic feasibility, and resource optimization. As a result, antihypertensive peptide development is evolving from a purely biochemical pursuit into an integrated strategy that addresses nutritional functionality, waste valorization, and sustainable food system design.

## 3. Materials and Methods

This systematic review was designed, conducted, and reported in accordance with the Preferred Reporting Items for Systematic Reviews and Meta-Analyses (PRISMA 2020) statement. The review question focused on the recent evidence describing the origin, mechanisms of action, molecular stability, bioavailability, and translational potential of food-derived antihypertensive peptides. The review followed a predefined screening and extraction workflow, and the main PRISMA elements relating to information sources, eligibility criteria, study selection, data items, synthesis methods, and study-flow reporting are presented below. Because this review synthesized published evidence, no new biological samples, experimental procedures, or proprietary datasets were generated.

### 3.1. Search Strategy and Information Sources

A comprehensive literature search was conducted in PubMed, Scopus, and Web of Science. Searches covered studies published from January 2020 through January 2025 and were limited to peer-reviewed articles written in English. The search strategy combined controlled vocabulary, when available, and free-text terms structured around three conceptual domains: (i) peptide and bioactivity terms; (ii) stability and bioavailability terms; and (iii) source terms. Search results were exported in standardized formats (CSV and RIS) for screening, deduplication, and data management.

- Peptide and Bioactivity Terms:
  “Bioactive peptides” OR “antihypertensive peptides” OR “ACE inhibitory peptides”
- Stability and Bioavailability Terms:
  “Stability” OR “bioavailability” OR “absorption” OR “transport” OR “gastrointestinal” OR “Caco-2”
- Source Terms:
  “Alternative source” OR “non-dairy” OR “non-meat” OR “plant protein” OR “insect protein” OR “algae” OR “seaweed”

**Figure 4:**
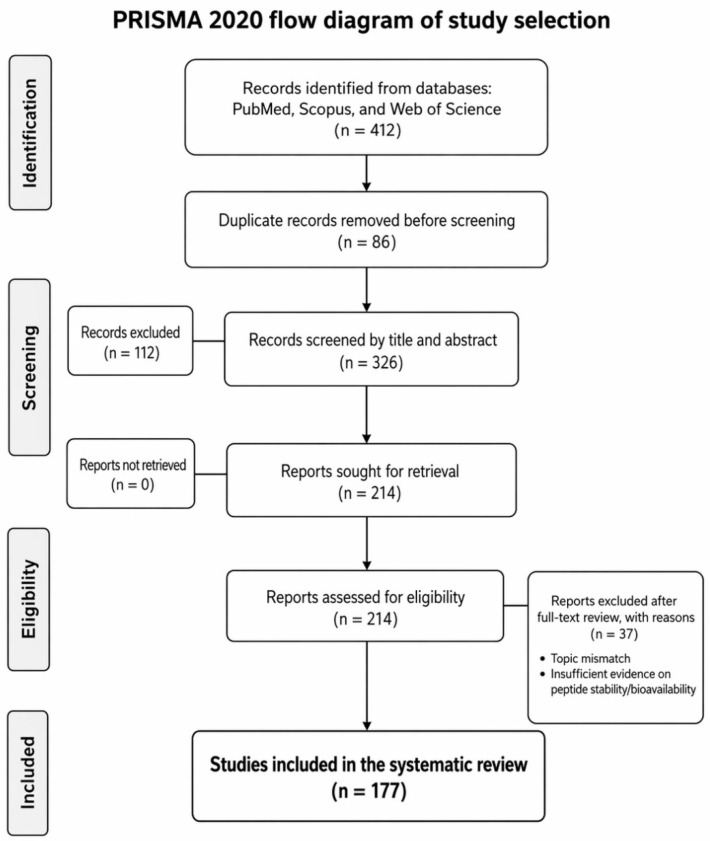
PRISMA 2020 flow diagram summarizing the identification, screening, eligibility assessment, and inclusion of studies in this systematic review.

The final Boolean strategy was adapted to the syntax requirements of each database. Manual checking of reference lists from highly relevant records was also performed to identify additional eligible studies. The complete study-selection process is summarized in the PRISMA flow diagram shown in Figure 4.

### 3.2. Inclusion Criteria

Studies were eligible when they met all of the following criteria: (1) original research articles reporting experimental data; (2) evaluation of food-derived peptides or peptide-rich hydrolysates with ACE-inhibitory and/or antihypertensive activity; (3) peptide origin from plant, marine, insect, fungal, dairy, egg, meat, or related food matrices, including coproducts and by-products; (4) reporting of at least one experimental dimension related to mechanism, molecular stability, gastrointestinal digestion, intestinal transport, bioavailability, vasodilatory response, or in vivo antihypertensive efficacy; and (5) publication in English between 2020 and 2025. Reviews, editorials, conference abstracts, patents, book chapters, and studies lacking direct experimental evidence on antihypertensive peptide activity were excluded. When several reports addressed overlapping peptide datasets, the most informative or complete report was prioritized.

### 3.3. Study Selection and Data Extraction

All retrieved references were imported into Microsoft Excel to create a master screening file. Duplicate records were removed before screening. Three reviewers independently screened titles and abstracts against the predefined eligibility criteria. Potentially relevant reports were then assessed in full text. Disagreements were resolved through discussion and consensus. For each included study, a standardized extraction framework was used to record source matrix, processing or hydrolysis approach, peptide sequences or fractions, analytical methods, in silico evidence, in vitro activity, ex vivo findings, in vivo outcomes, stability characteristics, bioavailability-related findings, and reported application in functional foods or nutraceutical systems.

The following data items were prioritized during extraction: protein source and food matrix; extraction, hydrolysis, fermentation, or fractionation procedures; peptide identity and molecular-weight characteristics; experimental models used; main antihypertensive mechanisms reported; indicators of gastrointestinal, thermal, processing, or storage stability; transport or absorption outcomes; in vivo efficacy indicators; and application-oriented findings related to delivery systems, sensory challenges, or industrial feasibility. Extracted information was cross-validated by the review team to improve completeness and consistency.

### 3.4. Synthesis Methods

Given the methodological heterogeneity of the included studies, findings were synthesized narratively rather than by meta-analysis. Studies were grouped according to food source, validation approach (in silico, in vitro, ex vivo, and in vivo), stability and bioavailability evidence, and functional food or nutraceutical application. Quantitative counts and proportions were used to summarize broad publication trends when these could be extracted consistently from the final study set.

### 3.5. Risk of Bias and Reporting Limitations

A formal tool-based risk-of-bias assessment was not performed because the objective of this review was to map methodological approaches and translational evidence across diverse experimental designs rather than to pool intervention effects. Nevertheless, the review considered recurrent reporting limitations during synthesis, including insufficient description of peptide purification, limited validation beyond in vitro ACE inhibition, heterogeneous digestion models, small animal-study sample sizes, and scarce human clinical evidence. These limitations were taken into account when interpreting the strength of the evidence.

### 3.6. PRISMA Flow and Study Yield

The screening workflow is summarized in Figure 4. After identification of records in PubMed, Scopus, and Web of Science, duplicate removal, title and abstract screening, and full-text eligibility assessment, 177 studies were retained for qualitative synthesis. This final number was used consistently throughout the revised manuscript.

### 3.7. Ethical Considerations

This review synthesizes previously published studies and did not involve human participants, animals, or new experimental interventions; therefore, ethical approval was not required. Studies included in the review that involved animal or human experimentation reported ethical approval from the corresponding institutional committees.

### 3.8. Use of Generative Artificial Intelligence (GenAI)

During manuscript preparation, ChatGPT (OpenAI, GPT-5.4 Thinking) was used only to support language refinement, organization of text, and editing assistance. The tool was not used to generate primary data, extract study results, determine study eligibility, or replace author judgment during screening, synthesis, or interpretation. All outputs were critically reviewed, corrected when necessary, and approved by the authors, who assume full responsibility for the final content.

## 4. Conclusions

This PRISMA-guided systematic review shows that another major advancement in the field is the widespread adoption of multistage evaluation frameworks integrating in silico, in vitro, ex vivo, and in vivo assays. This approach moves beyond traditional activity screening and enables comprehensive assessment of molecular stability, gastrointestinal resistance, absorption mechanisms, and antihypertensive efficacy in physiologically relevant models. Such methodologies are becoming essential for the translation of bioactive peptides into commercial applications, including nutraceutical formulations and functional foods. Moreover, nanoencapsulation is emerging as a transformative technology to overcome limitations of gastrointestinal degradation and poor intestinal permeability.

Despite this progress, several knowledge gaps remain. First, large-scale, randomized, controlled clinical trials are urgently needed to establish optimal dosages, long-term efficacy, and safety in human populations. Second, future research must prioritize standardization of processing and production methods, ensuring reproducibility and regulatory compliance. Addressing sensory challenges, such as bitterness, will also be critical to improving consumer acceptance of peptide-enriched functional foods. Finally, clearer regulatory pathways—including FDA GRAS notification and EU Novel Food authorization—are required to bridge the divide between preclinical validation and real-world market availability.

Overall, the growing body of evidence underscores the significant potential of antihypertensive peptides as sustainable, effective, and multifunctional ingredients within the global strategy to combat hypertension. Continued interdisciplinary research will be essential to unlock their full therapeutic and commercial value.

## Figures and Tables

**Table 1:** Summary of In Silico Tools and Their Applications in the Evaluation of Antihypertensive Peptides.

In Silico Assay	Purpose	Articles
Molecular Docking & Virtual Screening	Predict peptide-ACE binding and interaction	[1,27,28,29]
Simulated Gastrointestinal Digestion (SGID)	Assess peptide stability and fragment release	[9,25,31]
Bioactivity Prediction (PeptideRanker, BIOPEP)	Identify ACE/DPP-IV inhibitory motifs	[9,17,27]
Toxicity & Allergenicity Prediction (ToxinPred)	Ensure peptide for safety food applications	[9,27,30]
Drug-Likeness & ADME (SwissADME)	Evaluate bioavailability oral and pharmacokinetics	[9,18]

**Table 4:** Summary of In Vivo Methodologies Used to Evaluate Antihypertensive Peptides.

In vivo Assay	Purpose	Articles
Blood Pressure Measurement (SBP, DBP, MAP)	Confirms antihypertensive effect in animal models	[31,32,37]
Organ Coefficients & Body Weight	Evaluates systemic impact and safety	[31,38]
Kidney & Heart Histology	Assesses structural protection against hypertension-induced damage	[37,48]
RAS Modulation (ACE, Ang II, ACE2)	Determines mechanism of blood pressure regulation	[31,32]
Oxidative Stress Markers (LPO, SOD, GSH)	Measures antioxidant effect in vivo	[34]

## Data Availability

No new datasets were generated for this review. The search strategy, screening framework, and extracted summary information supporting the conclusions of this manuscript are available from the corresponding author upon reasonable request.
